# A Rare Case of Metastasis of Small Cell Carcinoma of Cervix to Breast

**DOI:** 10.14740/wjon858w

**Published:** 2015-02-14

**Authors:** Dhiraj Yadav, Siddhartha Yadav, Mohammad Muhsin Chisti

**Affiliations:** a Department of Internal Medicine, Beaumont Health System, 3601 W 13 Mile Rd, Royal Oak, MI 48073, USA; b Department of Hematology and Oncology, Oakland University William Beaumont School of Medicine, 43097 Woodward Ave, Bloomfield Hills, MI 48302, USA

**Keywords:** Small cell carcinoma, Cervix, Metastasis, Breast

## Abstract

Extrapulmonary small cell carcinomas (SCCs) are rare and often have an aggressive natural course. A 42-year-old female presented to the hospital with vaginal bleeding and lower abdominal pain. She was eventually diagnosed with SCC of cervix by biopsy. She was treated with chemoradiation. However, on follow-up positron emission tomography (PET) scan, fluorodeoxyglucose (FDG) uptake was noted in bilateral breasts. Biopsy of these lesions was consistent with metastatic SCC. Breast is a very unusual site for metastasis of cervical SCC and only four cases have been reported in the medical literature to date. Our case highlights the importance of considering metastatic disease when evaluating breast mass in patients with history of SCC of cervix.

## Introduction

Extrapulmonary small cell carcinomas (SCCs) are rare and often have an aggressive natural course. Although they frequently present with early metastases, breast tissue is an unusual site for metastasis of SCC.

## Case Report

**Figure 1:**
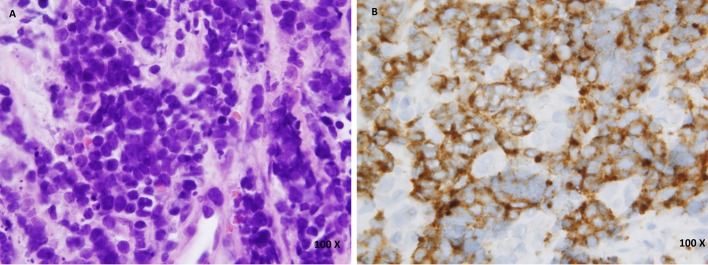
(A) Hematoxylin and eosin (H&amp;E) staining of cervical biopsy. (B) Synaptophysin staining of cervical biopsy.

A 42-year-old female presented to the hospital with vaginal bleeding and lower abdominal pain of 3 months duration. On pelvic exam, cervix was necrotic with oozing friable tissue extending to the pelvic wall consistent with stage IIIb cervical cancer. Biopsy of the cervical mass showed small cell neuroendocrine carcinoma with positive immunohistochemical staining for chromogranin and synaptophysin (Fig. 1).

Staging contrast enhanced CT scan of the chest, abdomen and pelvis showed a large heterogeneously enhancing cervical mass measuring 5.3 × 4.8 cm on axial imaging. There was a prominent right external iliac lymph node measuring 1 × 1.9 cm. Positron emission tomography (PET) scan was positive for malignancy at the cervical mass with suspicion of right external iliac node involvement with no abnormal fluorodeoxyglucose (FDG) uptake noted elsewhere.

**Figure 2:**
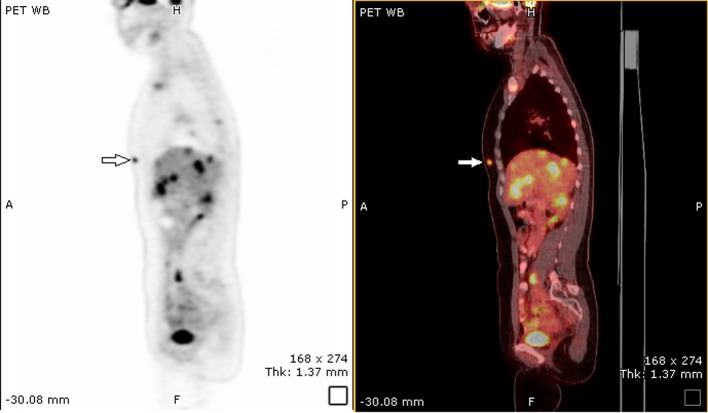
PET scan showing FDG uptake in right breast and liver.

She was treated with etoposide, cisplatin and radiation. Repeat CT scan after completion of chemoradiation showed significant interval reduction in size of cervical mass which now measured 3.4 × 2.7 cm. Previously identified right external iliac lymph node had resolved. On repeat whole body PET scan, there were two foci of activity in the breasts. On the left side, there was a focus of activity deep and superior to the left areola. On the right side, there was a focus of activity in the medial aspect of the right breast (Fig. 2). There was also evidence of multifocal metastatic disease involving the skeleton and liver.

**Figure 3:**
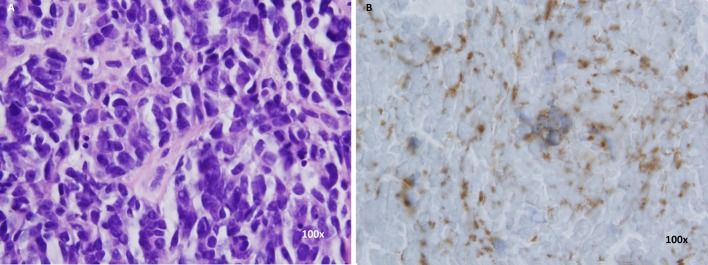
(A) H&amp;E staining of breast biopsy. (B) Synaptophysin staining of breast biopsy.

Biopsy of the right breast mass was done which showed small cell neuroendocrine carcinoma consistent with metastatic disease. Immunohistochemical staining for synaptophysin and chromogranin was positive (Fig. 3). The patient’s prior cervical biopsy was reviewed concurrently and the malignant cells present in that specimen were identical to the malignant cells seen in breast biopsy specimen. These cytomorphologic and immunohistochemical findings were felt to be consistent with metastatic small cell neuroendocrine carcinoma of the uterine cervix.

She received palliative radiation therapy to spine and liver. She had notable improvement in her back pain after a week of radiation therapy. Eventually, she was discharged with home hospice.

## Discussion

SCC of the cervix is an uncommon variant comprising about less than 2% of all cervical cancers and often diagnosed at a later stage compared to other types [1]. It demonstrates an aggressive behavior and metastasizes early to lymph nodes and distant organs indicating a poor prognosis [1-3]. Widespread dissemination can be seen involving bone, liver, lung, lymph nodes and other soft tissues [4], but involvement of breast has rarely been reported in the literature. Hsieh et al in 2011 reported a case of neuroendocrine tumor of cervix in a 46-year-old patient metastatic to adrenal gland and breast [5]. Similarly, Viswanathan et al also reported a case of recurrence of small cell cervical cancer in breast [4]. Extensive search of the literature revealed only isolated reports of breast metastasis in SCC of cervix [6, 7]. Other unusual sites of metastasis such as cerebellum and masseter have also been reported for SCC [8, 9].

SCC of cervix is diagnosed based on the histology and immunohistochemistry. Hematoxylin and eosin stains are similar to SCC of lung and other sites. Tumor is composed of poorly differentiated small blue cells which have hyperchromatic nuclei, scant cytoplasm, and inconspicuous nucleoli. Frequent mitoses and necrosis are also seen histologically. At least one of the neuroendocrine markers are present in about 88-100% cases [10]; however, their presence is not required for diagnosis. CT scan of the chest, abdomen and pelvis or a PET scan is warranted for all patients diagnosed with SCC [11]. Brain imaging is not required unless the patient has neurological symptoms or has pulmonary metastases [4, 11].

Treatment of cervical SCC is best done with a multimodality strategy and often involves surgery, chemotherapy and radiation. Hysterectomy may be required to secure diagnosis or in early diagnosed cases, as part of the multimodality therapeutic strategy [11]. Chemotherapy usually involves platinum-based regimen; radiation is given for those with locally advanced disease. Long-term survival can be achieved with this combined approach especially for those patients with disease limited to central pelvis [12]. Several clinicians have reported superior outcomes with use of chemotherapy in addition to local treatment [12, 13]. However the overall survival rate remains dismal, about 29% at 5 years [4]. Some of the poor prognostic factors are advanced stage, tumor size more than 2 cm, positive margins and history of smoking [6]. In one report, all patients who died of their disease had extrapelvic metastases with lung and liver being the most common sites [2]. This also signifies the importance of attempts at early detection of distant metastasis. Treatment should aim at local disease control and prevention of metastasis and many believe that it should conform to the treatment of SCC of lung because of similar natural history [14].

Our report presents a case of a rare though aggressive tumor with metastasis to an unusual site which was diagnosed with PET scan and pathological examination of the tissue. These diagnostic modalities are often essential in deciding further therapeutic strategies and also reflect the prognosis for a patient.
